# Delineating the impact of childhood traumatic brain injury (TBI) on long-term depressive symptom severity: Does sub-acute brain morphometry prospectively predict 2-year outcome?

**DOI:** 10.1016/j.nicl.2024.103565

**Published:** 2024-01-09

**Authors:** Nicholas P. Ryan, Dawn Koester, Louise Crossley, Edith Botchway, Stephen Hearps, Cathy Catroppa, Vicki Anderson

**Affiliations:** a School of Psychology, Deakin University, 221 Burwood Highway, Burwood 3125, Victoria, Australia; b Brain & Mind Research, Murdoch Children’s Research Institute, 50 Flemington Road, Parkville 3052, Victoria, Australia; c Psychology Service, Royal Children’s Hospital, Murdoch Children’s Research Institute, 50 Flemington Road, Parkville 3052, Victoria, Australia; d Department of Paediatrics, University of Melbourne, 50 Flemington Road, Parkville 3052, Victoria, Australia

**Keywords:** Traumatic brain injury, Childhood, Depression, Internalising, Neuroimaging

## Abstract

Despite evidence of a link between childhood TBI and heightened risk for depressive symptoms, very few studies have examined early risk factors that predict the presence and severity of post-injury depression beyond 1-year post injury. This longitudinal prospective study examined the effect of mild-severe childhood TBI on depressive symptom severity at 2-years post-injury. It also evaluated the potential role of sub-acute brain morphometry and executive function (EF) in prospectively predicting these long-term outcomes. The study involved 81 children and adolescents with TBI, and 40 age-and-sex matched typically developing (TD) controls. Participants underwent high-resolution structural magnetic resonance imaging (MRI) sub-acutely at five weeks post-injury (*M* = 5.55; *SD* = 3.05 weeks) and EF assessments were completed at 6-months post-injury. Compared to TD controls, the TBI group had significantly higher overall internalizing symptoms and were significantly more likely to exhibit clinically significant depressive symptoms at 2-year follow-up. The TBI group also displayed significantly lower EF and altered sub-acute brain morphometry in EF-related brain networks, including the default-mode network (DMN), salience network (SN) and central executive network (CEN). Mediation analyses revealed significant indirect effects of CEN morphometry on depression symptom severity, such that lower EF mediated the prospective association between altered CEN morphometry and higher depression symptoms in the TBI group. Parallel mediation analyses including grey matter morphometry of a non-EF brain network (i.e., the mentalising network) were not statistically significant, suggesting some model specificity. The findings indicate that screening for early neurostructural and neurocognitive risk factors may help identify children at elevated risk of depressive symptoms following TBI. For instance, children at greatest risk of post-injury depression symptoms could be identified based in part on neuroimaging of networks implicated in EF and post-acute assessments of executive function, which could support more effective allocation of limited intervention resources.

## Highlights

• Prospective study of factors linking childhood TBI to elevated depressive symptoms.
• We evaluated brain morphometry and executive function (EF) in predicting depression.
• Brain network morphometry differed between children with TBI, and healthy controls.
• We show an indirect effect of central-executive-network volume on depression via EF.
• Neurostructural and cognitive risk factors could help identify vulnerable children.

## 1 Introduction

Traumatic brain injury (TBI) is a leading cause of hospitalization of young people in Australia and globally (Menon et al., 2010). Recent estimates suggest that up to one third of children and adolescents with TBI will experience post-injury depressive symptoms, characterised by recurrent and chronic low mood, including feelings of worthlessness, anhedonia, withdrawal, and maladaptive thinking (Laliberté Durish et al., 2018). Despite evidence of a link between childhood TBI (cTBI) and heightened risk for post-injury depressive symptoms (Li and Liu, 2013), early risk factors that might help explain this association are poorly understood.

Compared to post-injury externalising behaviours that have been extensively studied into the long-term following childhood TBI (Li and Liu, 2013, Ryan et al., 2015), relatively fewer studies that have examined internalising behaviours such as depression. Drawing upon the limited research in this area, a recent scoping investigation found that 5.3 % to 36 % of children and adolescents with TBI exhibit post-injury depressive symptoms (Laliberté Durish et al., 2018). Similarly, when outcomes are examined categorically, it is estimated that 10–25 % of young people with TBI meet criteria for novel depressive disorders (Li and Liu, 2013). Despite the potential for post-injury depression symptoms to impact academic functioning, sleep, suicide risk, and quality of life (Laliberté Durish et al., 2018), there is a dearth of prospective outcome studies examining post-injury depressive symptoms beyond 1-year post injury. Consequently, knowledge of early risk factors to inform early preventive interventions for post-injury depressive symptoms in the child TBI population is lacking (Ryan et al., 2016).

Preliminary findings suggest that post-TBI depressive symptoms may be linked to a range of injury-related, pre-injury, and environmental factors. Laliberté Durish and colleagues (Laliberté Durish et al., 2018) found that higher post-injury depressive symptom severity is associated with family history of anxiety disorders and low socio-economic status (SES). Another study by Max and colleagues (Max et al., 2012) reported that frontal, temporal, and parietal cortical lesions were predictive of depression in mild-complicated to severe TBI. Based on these preliminary findings from a relatively small number of primarily retrospective studies, further prospective studies are needed to evaluate the potential role of early neurostructural and neurocognitive risk factors in predicting depressive symptoms that may persist in the longer-term beyond 1-year post-injury.

One possibility is that post-injury depressive symptoms are at least partly explained by structural damage to large-scale brain networks involved in executive function (EF). EF is a broad construct involving a range of inter-related cognitive processes involved in goal-directed behaviours (Levin and Hanten, 2005). EF is essential for daily functioning and involves a range of skills that are vulnerable to the effects of cTBI (Levin and Hanten, 2005, Anderson and Catroppa, 2005, Nadebaum et al., 2007, Sesma et al., 2008). These EF skills include *inhibition* (the ability to suppress automatic/habitual responses), *working memory* (the ability to temporarily maintain and manipulate information in conscious awareness) and *cognitive flexibility* (the ability to shift attentional focus between tasks and mental sets). These EF skills interact dynamically to support *emotional regulation* (Anderson, 2014), which describes an ability to use adaptive strategies to diffuse or moderate negative emotions and thoughts.

Neuroimaging studies indicate that EF is dependent on structural and functional connectivity of several large-scale brain networks in the developing brain (Engelhardt et al., 2019). These systems include the salience network (SN) anchored in the insula and anterior cingulate cortex, as well as the central executive network (CEN), which includes the dorsolateral prefrontal cortex, posterior parietal cortex, caudate nucleus, and thalamus (Dennis et al., 2013, Ryan et al., 2017). While the CEN is typically activated during externally oriented, goal-directed behaviour (Sherman et al., 2014); the SN supports EF through its role in modulating the anticorrelated network interactions of the CEN and the default mode network (DMN), which is anchored in the posterior cingulate cortex and the medial prefrontal cortex. Moreover, the DMN plays an indirect yet important role in higher-order cognitive processes (Chen *et al.*, 2013). For instance, higher-order cognition relies on the availability of neuronal resources, which is accomplished by diminishing activation of the DMN during top-down mental processing (Baum *et al.*, 2017, Gusnard *et al.*, 2001, Satterthwaite *et al.*, 2013).

Although childhood TBI is shown to involve abnormalities of large-scale brain networks implicated in EF (Dennis et al., 2013, Ryan et al., 2017), it remains unclear whether injury-related structural abnormalities of the CEN, DMN and SN may predict depressive symptom severity in the longer-term post-injury. According to one hypothesis, structural damage to the CEN, DMN, and/or SN may impair dynamic brain network interactions required to support flexible, goal directed behaviour. More specifically, structural damage to these networks may impair inhibitory control and cognitive flexibility, which may in turn contribute to excessive rumination and ineffective emotion regulation, including an inability to disengage from the biased, negative automatic thought patterns that underlie depression (Menon, 2011, Wang et al., 2016). This hypothesis is consistent with a broader literature showing that depression is commonly associated with executive dysfunction, including difficulties with inhibitory control and mental flexibility that may underlie the maintenance of biased, negative thought patterns and rumination in depression (Gotlib and Joormann, 2010).

Despite the well-documented association between executive dysfunction and depressive symptoms in the general population (Chahal et al., 2021, Snyder, 2013, Wagner et al., 2015, Halse et al., 2022); only a small number of studies have explored the role of EF as a potential risk factor in predicting depression following childhood TBI. In one study, Kurowski and colleagues (Kurowski et al., 2013) found that parent-ratings of executive dysfunction were associated with higher mood problems in adolescents with severe TBI. In another study, Ryan and colleagues (Ryan et al., 2021) reported that post-injury executive dysfunction mediated the relationship between abnormal brain structure and aggressive/rule-breaking behaviours at 12-months following childhood TBI. Given the putative involvement of EF in depression and the well-documented vulnerability of EF-related brain networks to damage from childhood TBI (Dennis et al., 2013, Hoskinson et al., 2019, Hayes et al., 2016), prospective studies are needed to evaluate whether post-injury executive dysfunction might mediate potential associations between structural abnormalities of the CEN, SN and/or DMN and long-term depressive symptoms in children with TBI.

### 1.1 Study aims

To address the relative dearth of literature regarding the nature and correlates of depressive symptoms in the long-term post injury, the first aim of this prospective cohort study was to evaluate the effect of childhood TBI on long-term depressive symptoms at 2-years post-injury in a relatively large sample of children and adolescents with medically confirmed TBI. Here, we hypothesised that children with TBI would exhibit significantly higher depressive symptoms than the age-and-sex matched typically developing control group.

The second study aim was to assess the potential role of sub-acute grey matter morphometry and executive functioning at 6-months post-injury in prospectively predicting long-term depression symptom severity at 2-years post-injury. We predicted that, compared to typically developing controls, children with TBI would display significant reductions in subacute CEN, SN and DMN grey matter volume, as well as significantly poorer EF assessed using parent-report surveys and performance-based EF measures at 6-months post-injury.

We also predicted that within the TBI group, poorer EF at 6-months post-injury would be prospectively associated with (i) smaller subacute CEN, SN and DMN volumes and (ii) increased depressive symptom severity at 2-years post-injury. Moreover, we predicted that poorer EF would mediate the prospective association between smaller subacute CEN/SN/DMN volumes and increased depressive symptom severity at 2-years post-injury.

## 2 Materials and methods

### 2.1 Participants

This is a longitudinal prospective study consisting of 121 children and adolescents, including participants with a diagnosis of TBI (*n* = 81) and typically developing controls (*n* = 40) recruited from local community schools chosen to ensure a range of sociodemographic backgrounds. Children with TBI were enrolled in the study at the time of injury and represented consecutive admissions to a Level 1 trauma center and emergency department in Melbourne, Australia (Anderson et al., 2017).

TBI group participants met the following inclusion criteria: (i) age 5–16 years at recruitment; (ii) documented evidence of closed head injury, including period of altered consciousness and two or more post-concussive symptoms; (iii) availability of medical records to enable determination of TBI severity based on Glasgow Coma Scale (GCS) (Teasdale and Jennett, 1974), neurological and radiological findings; (iv) no documented pre-injury neurological, developmental or social-emotional disorders, inflicted injury or previous TBI; (v) English language proficiency. Typically developing control children were required to meet criteria (i), (iv), and (v).

Children with TBI were further categorized as ‘mild TBI’ (GCS 13–15 with no evidence of mass lesion), ‘mild-complicated TBI’ (GCS 13–15 with mass lesion), “moderate TBI” (GCS 9–12; mass lesion/other injury and/or neurological impairment); and “severe TBI” (GCS 3–8; mass lesion/other injury and/or neurological impairment). Participants with TBI received routine post-injury care.

### 2.2 Measures

#### 2.2.1 Depressive symptom severity at 2-years post-injury

Parents completed the Child Behavior Checklist (CBCL), which is a well-validated and reliable parent-report measure used widely to screen for depression and anxiety symptoms in both TBI and non-clinical samples (Achenbach and Rescorla, 2001). The CBCL involves 113 items, which are rated on a 3-point Likert Scale (Achenbach and Rescorla, 2001) and used to derive age-adjusted T-scores (M = 50; SD = 10). For the current study, depressive symptom severity was measured using the well-validated, 8-item CBCL Withdrawn/Depressed subscale, which assesses depression symptoms characterized by sadness and loss of interest (e.g., unhappy; sad; depressed; withdrawn; shows little interest). We also employed the CBCL Internalizing Problems scale, which is a broadband measure of overall internalising symptoms.

#### 2.2.2 Executive function at 6 months post-injury

*Performance-based EF measures.* Subtests of the Tests of Everyday Attention for Children (TEA-Ch) (Manly et al., 2001) and Wechsler Intelligence Scale for Children-Fourth Edition (WISC-IV) (Wechsler, 2003) were used to measure EF skills in three domains (Inhibitory Control, Attention/Working Memory, and Cognitive Flexibility) in a controlled, standardized environment. TEA-Ch measures attentional skills of individuals aged 6–16 years. The WISC-IV measures general intelligence of individuals aged 6:0–16:11 years. TEA-Ch and WISC-IV subtests provide age-adjusted standard scores (M = 10; SD = 3). A Performance Composite, calculated by averaging the subtest scores from *TEA-Ch Walk/Don't Walk, TEA-Ch Creature Counting* and *WISC-IV Digit Span*, was deemed meaningful based on previous principal components analyses indicating that these three subtests load onto a single latent EF factor (Robinson et al., 2014).

*Parent-rated EF problems in daily life.* The parent-report version of the Behaviour Rating Inventory of Executive Functioning (BRIEF) was used to provide ecologically valid information concerning post-injury EF problems in everyday environments (Gioia et al., 2000). The BRIEF is an 86-item survey that quantifies the frequency and severity of everyday executive function difficulties using age-adjusted T-scores (M = 50; SD = 10). Higher scores denote greater EF problems in day-to-day life. In this study, we assessed everyday EF problems using the Behaviour Regulation Index (BRI). The BRI is a composite measure derived from three of the BRIEF subscales, including the Inhibit, Shift and Emotional Control subscales.

#### 2.2.3 Pre-Injury, injury and demographic variables

At initial recruitment, the primary caregiver retrospectively evaluated the child’s pre-injury functioning using the Child Behaviour Check List (Achenbach and Rescorla, 2001) and the Adaptive Behaviour Assessment System-II (ABAS-II) (Harrison and Oakland, 2003). The ratings were compared to TD controls who were age-matched to the TBI group. Family SES was determined using the Australian Socioeconomic Index (McMillan et al., 2009). General intelligence was measured at 6 months using the Weschler Abbreviated Scale of Intelligence (Wechsler, 1999). The injury characteristics of the TBI patients were extracted from the children’s electronic medical records using a standard clinical report form.

#### 2.2.4 Sub-acute brain morphometry

MR images were acquired at five weeks post-injury (M = 5.55; SD = 3.05 weeks) on a 3 Tesla Siemens Trio scanner (Siemens Medical Systems, Erlangen, Germany) using a 32-Channel matrix head coil. As per the protocol recommended by the National Institute of Neurological Disorders and Stroke Common Data Elements group, the MRI sequences included: T1- and T2-weighted, axial T2-weighted fluid attenuated inversion recovery (FLAIR), axial susceptibility weighted, and axial diffusion-weighted sequences. For each participant, T1‐weighted structural MR imaging was acquired using a three‐dimensional T1‐weighted magnetization‐prepared rapid gradient‐echo (MPRAGE) sequence with the following parameters: TR/TE = 1900/2.52 ms, FOV = 250 mm, isotropic voxel size = 1 mm.

Cortical reconstruction and volumetric segmentation were completed using FreeSurfer v7.1.1 (surfer.nmr.mgh.harvard.edu), which extracts whole brain volumetric measurements (i.e. total cortical, corpus callosum, white matter and grey matter volumes) and anatomic regions of interest using an algorithm that considers sulcal and gyral information. Volumetric measurements were based on the Desikan–Killiany atlas (Desikan et al., 2006), applied in the child’s own native space.

Pre-defined ROIs in FreeSurfer (Desikan et al., 2006) were obtained by extracting volumes for each ROI bilaterally. To evaluate our specific study hypotheses, network-based grey matter morphometry measures for individual subjects were derived by summing together grey matter volumetric values of bilateral ROIs within each of the respective neural networks of interest – CEN, SN and DMN (Dennis et al., 2013, Hoskinson et al., 2019). In keeping with existing literature (Dennis et al., 2013, Hoskinson et al., 2019), the CEN composite includes the dorsolateral prefrontal cortex, posterior parietal cortex, caudate nucleus, and thalamus, whereas the SN composite includes the ventrolateral prefrontal cortex, insula, anterior cingulate cortex, and amygdala. Similarly, the DMN composite was derived by summing the volumes of the bilateral ventromedial prefrontal cortex (vmPFC), posterior cingulate cortex (PCC), inferior parietal lobule (IPL), and hippocampal formation (HF). This process yielded three network-based morphometry variables (i.e., brain morphometry values for the DMN, CEN and SN, respectively) for use in subsequent analyses. To evaluate the relative specificity of the models, we conducted parallel analyses that involved substituting the neural networks of interest (DMN, CEN and SN) with a non-EF network implicated in social information processing. Specifically, the Mentalizing Network (MN) grey matter composite included the bilateral caudal and rostral middle frontal cortex (i.e., the dorsomedial prefrontal cortex), superior temporal gyrus, bank of the superior temporal sulcus, middle temporal gyrus, supramarginal gyrus and temporal pole.

### 2.3 Procedure

The RCH Human Research Ethics Committee and Victorian Department of Education Ethics Committee, Melbourne, Australia approved the study. Parents gave written, informed consent; verbal consent was sought from participants aged over 8 years. Research MRI scans were obtained at five weeks post-injury (*M* = 5.55; *SD* = 3.05 weeks). Cognitive and social-behavioural evaluations were obtained at 6-months and 2-years post-injury. Psychometric tests were administered in person by a trained child psychologist.

### 2.4 Statistical analysis

Statistical analyses were conducted using SPSS Statistics 28 (IBM Corporation). Between group differences on sample demographic and injury characteristics were analysed using one-way analysis of variance (ANOVA) for continuous variables and Chi-square for categorical variables. Independent samples *t*-tests were conducted to examine differences between TBI and TD control groups on depressive symptom severity at 2-years post-injury, and EF at 6 months post-injury. One-way analyses of covariance (ANCOVAs) with Bonferroni-corrected post-hoc pairwise comparisons were used to evaluate the effect of TBI severity group membership on measures of network-based grey matter morphometry (CEN, SN, DMN), after adjusting for child sex, age, socio-economic status and total intracranial volume.

Mediation analyses were conducted using PROCESS V2.16 for SPSS. In the statistical literature, mediation is quantified by the effect of the independent variable (*X*) on the dependent variable (*Y*) through the proposed mediator (*M*), which is called the *indirect effect*. In this study, mediation analyses were run with bootstrap resampling, which is a powerful method in detecting indirect effects with no assumptions required of the sampling distribution for these effects. Bootstrapping produces upper and lower bound confidence intervals for the point estimate of an indirect effect, which should exclude 0 to reject the null hypothesis. PROCESS mediation analyses were conducted using model 4 and bias-corrected 95 % confidence intervals (CIs) produced from 10,000 bootstrap resamples.

Although traditional approaches to mediation indicate that a direct association between *X* and *Y* is a necessary pre-condition to proceed with mediation analysis, more recent statistical approaches allow potential indirect effects to be evaluated when variables of interest meet two specific preconditions, including (i) a significant association between *X* and *M*, and (ii) a significant association between *M* and *Y* (Rockwood et al., 2020, Hayes, 2009). Accordingly, and to identify measures of brain morphometry, EF and symptom severity that met criteria for inclusion in subsequent mediation models (Hayes, 2009), simple bivariate associations between variables were assessed using Pearson correlations: for correlations involving brain morphometry measures, age at MRI and estimated total intracranial volume (ICV) were includes as covariates. Using those measures that met the statistical preconditions for mediation (Hayes, 2009), we constructed a series of mediation models to evaluate hypothesized indirect effects of brain morphometry on depression via EF. For each model, brain morphometry was entered as an independent variable, and EF was entered simultaneously as the proposed mediator. SES, child sex, TBI severity, pre-injury internalising symptoms, age at MRI and total intracranial volume were included as covariates in each mediation model.

## 3 Results

### 3.1 Sample characteristics

Table 1, Table 2 display the demographic and injury characteristics of the sample, including the TD control group (*n* = 40) and children with mild, mild complicated, moderate, and severe TBI (*n* = 81). All groups were comparable on age and sex.

**Table 1:** Sample demographic characteristics.

	TD control	Mild TBI	Mild-Complicated TBI	Moderate TBI	Severe TBI	*F*/ *χ*^2^	*p*
Male, *n* (%)	24 (55.8)	44 (77.2)	8 (57.1)	16 (61.5)	8 (53.3)	6.68	0.154
Recruitment age (years), *M(SD)*	10.25 (3.04)	10.67 (2.36)	9.47 (2.44)	10.33 (2.49)	9.72 (3.01)	0.80	0.528
MRI age (years), *M (SD)*	10.41 (2.76)	10.80 (2.33)	9.57 (2.43)	10.37 (2.58)	10.41 (3.10)	0.66	0.621
Socio-economic status, *M(SD)*	76.75 (14.19)	70.17 (19.64)	58.61 (27.09)	60.88 (24.76)	70.66 (25.09)	3.44	0.010
WASI FSIQ, *M(SD)*	106.81 (15.20)	99.96 (16.92)	95.14 (17.04)	93.13 (18.79)	92.33 (17.63)	3.68	0.007
Pre-injury ABAS Social, *M(SD)*	10.44 (2.74)	10.02 (3.37)	9.46 (3.20)	10.00 (2.08)	9.86 (3.42)	0.32	0.862
Pre-injury ABAS Adaptive Composite, *M(SD)*	97.37 (15.38)	97.26 (17.15)	95.77 (16.15)	98.32 (13.08)	94.71 (15.75)	0.14	0.965
Pre-injury CBCL Internalizing	47.16 (8.31)	48.26 (11.25)	51.54 (12.33)	47.88 (9.58)	49.21 (9.58)	0.50	0.733
Pre-injury CBCL Externalizing	46.63 (6.99)	48.13 (10.09)	51.39 (10.40)	48.04 (11.23)	50.50 (9.88)	0.86	0.488

*Note*. TD = typically developing; WASI FSIQ = Weschler Abbreviated Scale of Intelligence - Full Scale Intelligence Quotient; ABAS = Adaptive Behaviour Assessment System.

**Table 2:** Injury characteristics and global brain volumes of the overall sample.

	TD Control	Mild TBI	Mild Complicated TBI	Moderate TBI	Severe TBI	*F*/ *χ*^2^	*p*
Age at injury (y), *M* (*SD*)	–	10.67 (2.36)	9.47 (2.44)	10.33 (2.49)	9.72 (3.01)	1.20	0.312
Lowest GCS, *M* (*SD*)	–	14.54 (1.04)	13.93 (1.14)	11.50 (1.98)	5.60 (2.20)	148.99	<0.001
Hospital stay (days), *M* (*SD*)	–	0.477 (0.73)	2.30 (1.82)	4.75 (4.59)	17.7 (13.94)	38.83	<0.001
Surgical intervention, *n (%)*	–	0 (0)	0 (0)	8 (30.8)	7 (46.7)	32.07	<0.001
Injury cause, *n (%)*						24.41	<0.001
MVA	–	4 (7.0)	2 (14.3)	10 (38.5)	9 (60.0)		
Fall/blow	–	53 (93.0)	12 (85.7)	16 (61.5)	6 (40.0)		
MRI pathology, *n (%)*							
Frontal	0 (0)	13 (23.6)	4 (28.6)	14 (56.0)	10 (76.9)	27.46	<0.001
Extra-frontal	0 (0)	6 (10.9)	4 (28.6)	9 (36.0)	6 (46.2)	20.56	<0.001
Sub-cortical	0 (0)	2 (3.6)	1 (7.1)	4 (16.0)	4 (30.8)	16.27	0.003
Global brain morphometry							
Tot. brain vol. *cm^3^*, *M* (*SD*)	1232 (1 1 8)	1262 (1 1 6)	1204 (86)	1222 (1 1 3)	1175 (1 0 3)	1.752	0.142
Tot. intracranial vol. *cm^3^*, *M* (*SD*)	1502 (1 3 8)	1536 (1 4 4)	1455 (1 1 9)	1500 (1 2 1)	1485 (1 8 0)	0.731	0.573
White matter total, *cm^3^*, *M* (*SD*)	435 (49.91)	439 (52.54)	415 (45)	433 (37)	427 (74)	0.349	0.845
Gray matter total, *cm^3^*, *M* (*SD*)	755 (71)	779 (65)	748 (44)	750 (88)	696 (59)^a,b^	3.929	0.005

GCS = Glasgow Coma Score; MVA = motor vehicle accident (passenger, cyclist, pedestrian); tot. = total; vol. = volume. Statistically significant Bonferroni-corrected post-hoc analyses comparing ^a^Mild TBI vs. Severe TBI, ^b^Moderate TBI vs. Severe TBI.

There were significant group differences in family SES, such that children with moderate TBI had lower SES than the TD control group. As expected, groups differed on general intellectual ability, such that estimated Full-Scale IQ scores were significantly lower in the severe TBI group than TD controls (Table 1). As expected, the TBI severity groups showed significant differences on injury-related variables, including lowest GCS (Table 2).

As shown in Table 1, no significant group differences were identified when the pre-injury adaptive function of the TBI groups was compared to adaptive function ratings of the TD control group collected at time of recruitment. Similarly, the TBI groups did not significantly differ from the TD control group on pre-injury externalizing symptoms or pre-injury internalizing symptoms on the Child Behaviour Check List (see Table 1).

### 3.2 Impact of TBI on depressive symptom severity and executive functioning

*Effect of overall group membership (TBI* vs*. TD control).* On the CBCL Internalising Problems broadband scale, TBI participants had significantly higher overall internalizing symptoms than the TD control group (see Table 3). Although the TBI group also had higher mean scores on the CBCL Withdrawn/Depressed Scale than the TD control group, the group difference did not reach statistical significance. However, when these same outcomes were examined categorically using established clinical cut off values (*T* > 65), the TBI group were significantly more likely than TD controls to exhibit depressive symptoms in the clinically elevated range on the CBCL Withdrawn/Depressed subscale (*p* =.019).

**Table 3:** Group means and standard deviations for executive function and depression symptom severity.

	TBI	TD control	*F*	*p*	*η2*
*EF measures – 6 months post-injury*					
BRIEF BRI, *M (SD)*	48.64 (11.92)	45.63 (6.37)	3.00	0.09	0.022
EF Composite, *M (SD)*	9.34 (1.99)	10.24 (1.91)	6.74	0.01*	0.045
*Depression severity – 2-years post-injury*					
CBCL Withdrawn-Depressed, *M (SD)*	52.30 (4.59)	51.53 (2.55)	0.98	0.33	0.008
CBCL Internalizing Total, *M (SD)*	46.43 (10.78)	43.68 (8.25)	4.35	0.03*	0.036

*Denotes statistically significant group difference, *p* <.05.

EF = Executive Function; BRIEF BRI = Behaviour Rating Inventory of Executive Function – Behaviour Regulation Index; CBCL = Child Behaviour Checklist.

As shown in Table 3, the TBI group also had significantly lower scores than the TD control group on the EF performance-based composite. With respect to everyday EF skills, the TBI group had more frequent parent-rated EF problems than TD controls on the BRIEF Behaviour Regulation Index (BRI); however, the difference did not reach statistical significance (see Table 3).

*Effect of TBI severity group membership (mild TBI* vs*. mild-complicated* vs*. moderate* vs*. severe TBI).* After correcting for age, sex, family socio-economic status and pre-injury internalising symptoms, ANCOVAs found no statistically significant main effect of TBI severity group membership on CBCL Internalising Problems (*F* = 1.32, *p* =.269, *η^2^* = 0.04), CBCL Withdrawn/Depressed symptoms (*F* = 0.64, *p* =.636, *η^2^* = 0.02), BRIEF BRI (*F* = 1.65, *p* =.166, *η^2^* = 0.05), or the EF performance-based composite (*F* = 1.98, *p* =.101, *η^2^* = 0.05).

### 3.3 Impact of childhood TBI on sub-acute network-based morphometry

For measures of global brain morphometry presented in Table 2, groups did not significantly differ on estimated total intracranial volume (ICV), total brain volume or total white matter volume.

Table 4 presents the mean grey matter volumes of the CEN, SN, and DMN, as well as the grey matter volumes of the individual regions of interest (ROIs) associated with these networks. After adjusting for participant age, sex, socio-economic status and total ICV, significant overall group differences were documented for CEN, SN and DMN morphometry. As shown in Table 4, post-hoc pairwise comparisons demonstrated that children with severe TBI had significantly smaller CEN, SN and DMN volumes than the TD control group and all other TBI severity groups, including the mild, complicated mild, and moderate TBI groups (all *p* <.05).

**Table 4:** Effect of TBI on sub-acute central executive network (CEN), salience network (SN) and default-mode network (DMN) morphometry.

Grey matter volume cm^3^ (SD)	TD Control	Mild TBI	Mild-Complicated TBI	Moderate TBI	Severe TBI	*F*	*p*	**η^2^**
**CEN**	171.48 (17.42)	177.11 (16.40)	172.50 (12.59)	174.45 (17.34)	158.44 (18.63)^a,b,c,d^	4.36	0.002	0.12
Dorsolateral PFC	83.69 (10.48)	88.40 (10.15)	85.14 (7.23)	86.55 (9.61)	77.56 (11.31)^a,b,c,d^	3.88	0.005	0.11
Posterior parietal cortex	63.71 (6.22)	64.25 (6.11)	63.87 (5.68)	64.32 (7.74)	58.77 (7.26)^a,b,c,d^	2.46	0.049	0.07
Caudate Nucleus	8.09 (1.33)	8.25 (1.07)	7.98 (0.98)	7.78 (0.82)	7.46 (0.79)^b^	1.47	0.214	0.04
Thalamus	15.99 (1.47)	16.20 (1.46)	15.51 (1.23)	15.81 (1.33)	14.65 (1.99)^a,b,c,d^	3.54	0.009	0.10
**SN**	52.55 (5.53)	54.00 (5.30)	51.84 (3.68)	53.01 (5.66)	48.56 (3.12)^a,b,c,d^	4.17	0.003	0.12
Ventrolateral PFC	3.73 (0.66)	3.83 (0.63)	3.81 (0.56)	3.85 (0.63)	3.26 (0.54)^a,b,c,d^	2.28	0.064	0.07
Insula	26.47 (2.92)	27.30 (2.92)	26.23 (2.01)	26.89 (3.01)	25.39 (1.75)^b^	1.25	0.293	0.04
Anterior cingulate cortex	19.09 (2.20)	19.48 (2.30)	18.73 (1.88)	19.02 (2.60)	16.98 (2.44)^a,b,c,d^	3.61	0.008	0.10
Amygdala	3.25 (0.44)	3.39 (0.40)	3.07 (0.34)	3.25 (0.46)	2.93 (0.47)^a,b,d^	3.10	0.018	0.09
**DMN**	116.50 (10.89)	118.68 (10.20)	114.68 (8.16)	117.50 (11.39)	103.64 (12.23) ^a, b, c, d^	7.83	<0.001	0.20
Ventromedial prefrontal cortex	40.00 (4.39)	41.42 (3.83)	39.25 (2.19)^f^	40.80 (3.77)	34.91 (5.79)^a, b, c, d^	8.60	<0.001	0.21
Posterior cingulate cortex	17.38 (2.31)	17.71 (2.36)	16.55 (1.56)	17.33 (2.43)	15.35 (2.89)^a,b,c,d^	3.11	0.018	0.09
Inferior Parietal Lobule	50.20 (5.01)	50.54 (5.07)	50.26 (4.66)	50.41 (5.63)	45.66 (5.44)^a,b,c,d^	3.15	0.017	0.09
Hippocampal Formation	8.92 (0.86)	9.02 (0.74)	8.61 (0.81)	8.97 (0.88)	7.72 (1.68)^a,b,c,d^	5.65	<0.001	0.15

Note: Significant (*p* <.05) Bonferroni corrected post-hoc differences: ^a^ TD vs. TBI-Severe, ^b^ TBI-Mild vs. TBI-Severe, ^c^ TBI-Complicated Mild vs. TBI-Severe, ^d^ TBI-Moderate vs. TBI-Severe, ^e^ TD vs. TBI-Moderate, ^f^TBI Mild vs. TBI-Complicated Mild.

PFC = prefrontal cortex; TD = typically developing.

### 3.4 Prospective associations among network-based morphometry measures, executive function, and depressive symptom severity at 2-years post-injury

Although traditional approaches to mediation indicate that a direct association between *X* and *Y* is a necessary pre-condition for mediation analysis, more recent statistical approaches require two specific preconditions to be met. This includes (i) a significant association between *X* and *M*, and (ii) a significant association between *M* and *Y* (Rockwood et al., 2020, Hayes, 2009). To identify those measures of brain morphometry, EF and symptom severity that met these preconditions (Hayes, 2009) simple bivariate associations were assessed using Pearson correlations: for correlations involving brain morphometry measures, age at MRI and estimated total intracranial volume (ICV) were entered as covariates.

As shown in Table 5, smaller CEN and DMN volumes were both significantly associated with higher symptom burden on the CBCL Withdrawn/Depressed Scale, after adjustment for age and estimated total ICV. Smaller CEN volume was also associated with poorer performance on the EF composite and higher parent-rated EF problems on the BRIEF BRI. Higher parent-rated EF problems and poorer performance on the EF composite were also associated with higher depressive symptom burden.

**Table 5:** Associations among network-based brain morphometry, executive function, and depressive symptom severity in the TBI group, *r (p)*.

	EF Composite	BRIEF BRI	CBCL IP Scale	CBCL W/D Scale
CEN morphometry	0.25 (0.035)	-0.26 (0.030)	-0.17 (0.160)	-0.25 (0.036)
SN morphometry	0.20 (0.095)	-0.18 (0.132)	-0.18 (0.136)	-0.23 (0.053)
DMN morphometry	0.12 (0.234)	-0.12 (0.263)	-0.18 (0.137)	-0.26 (0.027)
EF Composite	–	-0.26 (0.032)	-0.39 (<0.001)	-0.41 (<0.001)
BRIEF Behaviour Regulation Index (BRI)	–	–	0.54 (<0.001)	0.34 (0.004)

*Note*. CBCL IP Scale = Child Behavior Checklist Internalizing Problems Scale; CBCL W/D Scale = Child Behavior Checklist Withdrawn/Depressed Scale; CEN = Central Executive Network, SN = Salience Network; EF, Executive Function.

SN morphometry was not significantly associated with any of the EF or depression symptom-based measures (Table 5).

### 3.5 Indirect effects of network-based brain morphometry on depressive symptom severity at 2-years post-injury via executive function

In keeping with study hypotheses, a series of mediation models were constructed to evaluate potential indirect effects of DMN and CEN morphometry on depressive symptom severity via executive function as the hypothesised mediator. All mediation models involved adjustment for relevant covariates, including TBI severity, sex, pre-injury internalising symptoms, age at MRI and total intracranial volume.

*Indirect effect of DMN morphometry*. Contrary to expectations, there were no significant indirect effects in models that included DMN morphometry (X), depression symptom severity (Y), and measures of child and parent-rated EF as the hypothesised mediators (M). A summary of the results for each of these models is provided in Supplementary Table S1.

**Fig. 1:**
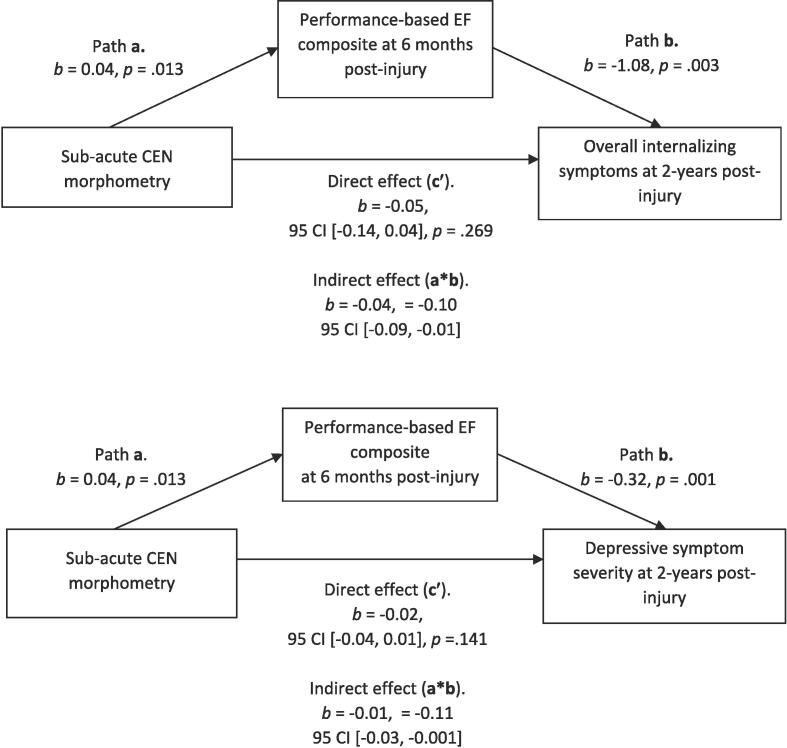
Indirect effect of CEN morphometry on post-injury depressive symptom severity and overall internalising symptoms, mediated by the performance-based EF composite.

*Indirect effect of CEN morphometry.* In Fig. 1, we evaluated the indirect effect of CEN morphometry on depressive symptom severity and overall internalising symptoms when the performance-based EF composite was entered as the proposed mediator in each model. As hypothesised, we found a significant indirect effect of sub-acute CEN morphometry on depressive symptom severity at 2-years post-injury, such that lower EF at 6-months post-injury mediated the prospective association between smaller CEN volume and increased depressive symptom severity and overall internalising symptoms (see Fig. 1).

**Fig. 2:**
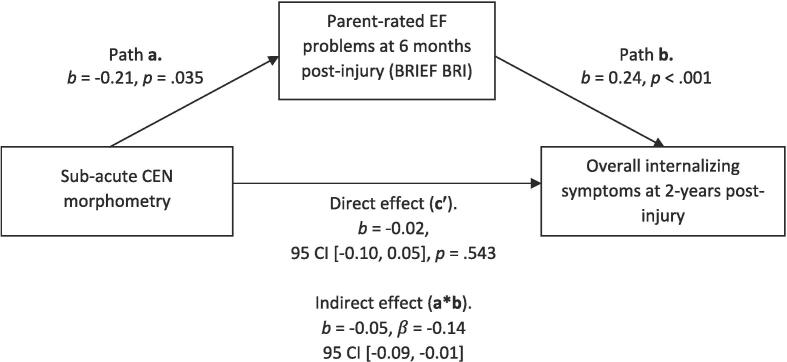
Indirect effect of CEN morphometry on overall internalising symptom severity, mediated by parent-rated EF problems on the BRIEF BRI.

**Fig. 3:**
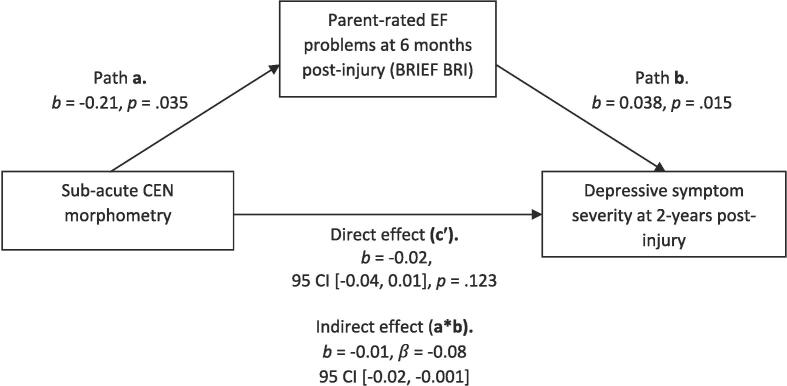
Indirect effect of CEN morphometry on post-injury depressive symptom severity, mediated by parent-rated EF problems on the BRIEF BRI.

In Fig. 2, Fig. 3, we assessed the indirect effect of sub-acute CEN morphometry on depressive symptom severity when parent-rated EF problems (as measured by BRIEF BRI) were entered as the proposed mediator in each model. As expected, we found a significant indirect effect of subacute CEN morphometry on overall internalising symptoms (Fig. 2) and depressive symptom severity at 2-years post-injury (Fig. 3), such that more frequent EF problems mediated the prospective association between smaller subacute CEN volume and increased symptom severity at 2-years post-injury.

### 3.6 Sensitivity analyses

To determine if significant findings were driven by children with severe TBI, the mediation models were re-run after excluding children with severe TBI. In keeping with the findings of the primary analyses reported above, all results remained highly similar when children with severe TBI were excluded (see Supplementary Table 2 (S2), available online).

### 3.7 Parallel analyses to assess relative specificity of indirect effects of CEN morphometry on depression symptom severity

To assess the relative specificity of the indirect effect of the CEN on depression severity via EF, we ran parallel mediation models in which the CEN was replaced by mentalising network (MN) morphometry. The MN is widely considered a non-EF network that is implicated in social information processing. Across each of these parallel models, we found no significant indirect effect of MN morphometry on depression severity via EF, suggesting some model specificity. The results of these parallel analyses are summarised in Supplemental Table 1 (S1), available online.

## 4 Discussion

Despite evidence of a link between childhood TBI and heightened risk for depressive symptoms (Laliberté Durish et al., 2018, Li and Liu, 2013), very few prospective studies have examined early risk factors that predict the presence and severity of post-injury depression beyond 1-year post injury. To address this gap in knowledge, this longitudinal prospective study examined the effect of mild-severe childhood TBI on parent-ratings of depressive symptom severity at 2-years post-injury. The study also evaluated the potential role of sub-acute brain morphometry and executive function in prospectively predicting these long-term outcomes at 2-year follow-up. As expected, the TBI group had significantly higher overall internalizing symptoms and was significantly more likely to exhibit clinically elevated depressive symptoms than the TD controls at 2-years post-injury. The TBI group also displayed significantly lower EF at 6-months post-injury, and these lower EF ratings mediated the prospective association between altered sub-acute CEN morphometry and higher depressive symptom severity at 2-years post-injury. Parallel mediation analyses that included grey matter morphometry of a non-EF brain network (i.e. the mentalising network) were not statistically significant, suggesting some model specificity.

At 2-years post-injury, the TBI group exhibited significantly higher depressive symptom burden, including significantly higher rates of clinically significant depressive symptoms than TD controls. This finding is consistent with prior research demonstrating that young people with TBI are at elevated risk for post-injury depressive symptoms (Laliberté Durish et al., 2018, Li and Liu, 2013). In addressing some limitations of previous research that has typically examined post-injury depression symptoms in relatively small samples of retrospectively recruited TBI patients, our results extend existing literature in showing that a larger, prospectively recruited cohort of children with TBI display elevated risk for post-injury depressive symptoms until at least 2-years post-injury. Given our prospective study methods involved exclusion of TBI participants with pre-injury psychiatric diagnoses (including major depression), we suggest that post-injury depression symptoms in our TBI sample are unlikely to be attributable to pre-injury depression.

Our results provided no evidence of a dose–response relationship between TBI severity and the frequency of depressive symptomology at 2-years post-injury. Of note, these findings contrast with results from Chapman and colleagues (Chapman et al., 2010), who found that severely injured children had higher internalizing problems than those with moderate TBI. Several factors might help to explain limited evidence for a linear dose–response relationship between TBI severity and depressive symptom severity in our TBI sample. Firstly, impairments in self-awareness are relatively common in severe brain injury and may act as a protective factor against depressive symptoms (Lloyd et al., 2021). Secondly, clinical indicators of TBI severity (including Glasgow Coma Scale scores) are relatively imprecise measures, which cannot accurately quantify the nature and extent of underlying brain tissue damage to neural networks implicated in EF.

As hypothesized, the study demonstrated that, relative to TD controls and those with mild-moderate TBI, participants with severe TBI displayed significantly smaller sub-acute CEN, DMN, and SN volumes. The results were expected based on previous findings (Ryan et al., 2017), and align with previous literature showing that the biomechanical forces of severe TBI are associated with disruption to brain networks involved in higher-order cognition (Zamani et al., 2020). Given we found no significant group differences in subacute total intracranial volume, these findings support the presence of regionally specific structural brain abnormalities in the sub-acute injury period.

cTBI commonly involves injury to prefrontal-subcortical brain circuits, and the importance of these brain circuits for the development of EF skills is well-established (Levin and Hanten, 2005, Zamani et al., 2020). As expected, performance-based and parent-rated EF measures each revealed that, on average, the TBI group displayed significantly poorer EF compared to TD controls at 6 months post-injury. The results supported prior research findings on post-injury EF deficits in children and adolescents with TBI (Anderson and Catroppa, 2005, Nadebaum et al., 2007). Of note, analysis of group means for performance-based EF measures showed larger effect sizes for cognitive flexibility than other EF domains. Again, this is broadly consistent with prior research, which indicates that brain regions involved in cognitive flexibility (e.g., medial prefrontal structures) may be selectively vulnerable to the effects of cTBI (Zamani et al., 2020).

In keeping with our predictions, poorer scores on both parent-rated and performance-based measures of EF were associated with smaller sub-acute CEN volumes. This result is consistent with the documented role of the CEN in EF development (Dennis et al., 2013), and suggest that altered CEN volume may be an early risk factor for executive dysfunction at 6-months post-injury. In keeping with predictions, lower EF was also prospectively associated with higher depressive symptomology at 2-years post-injury. Evidence for a strong, prospective relationship between lower EF and greater depressive symptom severity aligns with prior literature regarding the role of cognitive dysfunction in depression in the general population (Halse et al., 2022, Yang et al., 2022), and suggests that post-injury executive dysfunction may be an important risk factor for long-term depressive symptoms in the cTBI population.

Based on prior research showing that depression is associated with structural and functional aberrations of large-scale neural networks implicated in EF (Wang et al., 2016, Kaiser et al., 2015, Jiang et al., 2017, Wang et al., 2020), we hypothesized that diminished CEN volume would indirectly predict higher depressive symptoms via its effect on post-injury EF. In keeping with this initial prediction, poorer EF was found to mediate the prospective association between altered sub-acute CEN morphometry and higher depressive symptoms at 2-years post-injury. Based on these results, we speculate that structural damage to the CEN may disrupt inter-regional brain connectivity required for flexible, top-down executive control of emotional responses and affective states. In this context, structural damage to the CEN may impair inhibitory control and flexible goal-directed thinking, which may in turn contribute to difficulties with modulating negative emotional responses and disengaging from the biased, negative automatic thought patterns that underlie depression (Menon, 2011, Wang et al., 2016). Overall, our study findings converge to suggest that post-injury executive dysfunction is an important risk factor for long-term depressive symptoms and may at least partly underlie the prospective relationship between sub-acute brain structural abnormalities and greater depression symptom severity in children with TBI.

### 4.1 Study implications

In a recent scoping review on this topic, Laliberté Durish and colleagues (Laliberté Durish et al., 2018) argue that both injury and non-injury related risk factors may play an important role in depression symptoms following childhood TBI. Extending on these findings, our study offers support for an indirect contribution of structural brain pathology to post-injury depressive symptoms via its influence on EF and underscore the potential value of subacute, high-resolution structural MRI in prospectively predicting post-injury EF outcomes. Specifically, findings suggest that sMRI abnormalities may aid early identification of children at elevated risk for post-injury executive dysfunction, which may in turn predict post-injury depression symptoms.

This study also highlights the potential benefits of integrating EF evaluation into routine screening of young people following TBI, and pre-emptive behavioural monitoring of cTBI patients with identified neurostructural and/or EF risk factors. The study may also have implications for design of preventive interventions targeting modifiable risk factors for depressive symptoms. Such preventive interventions may incorporate psychological strategies addressing emotional regulation, cognitive flexibility, and inhibition. Such interventions may in turn help reduce the likelihood of post-injury depression.

### 4.2 Study limitations and future directions

This study is not without methodological limitations. Firstly, this study analysed structural brain MRI data obtained at a single time-point. As such, these data cannot be used to establish causal relationships between the variables. Secondly, this study aimed to evaluate only the role of neurostructural and neurocognitive risk factors in prospectively predicting post-injury depressive symptoms, after adjustment for child age, sex, total intracranial volume, family socioeconomic status and pre-injury internalising symptoms. Given our exclusive focus on neurostructural and neurocognitive variables in outcome prediction, future research is needed to examine the independent contribution of other non-injury related risk factors, including family functioning and parent mental health (Zamani et al., 2020).

Thirdly, we cannot entirely rule out the possibility that depressive symptoms in our TBI sample are at least partly explained by pre-injury psychiatric vulnerabilities, including symptoms of anxiety and depression that pre-dated the injury. Nevertheless, there are several factors that do not support this possibility. Firstly, retrospective ratings of pre-injury functioning showed that the TBI groups did not significantly differ from typically developing controls on measures of pre-injury externalizing symptoms, adaptive behaviour, or internalizing symptoms. Viewed collectively with evidence showing that all findings remained statistically significant after adjusting for pre-injury internalizing symptoms, our results suggest that post-injury depressive symptoms in our TBI sample are more likely related to brain injury-related processes than premorbid psychiatric vulnerabilities.

Further research is needed to employ a broader range of MRI analytic techniques (e.g., voxel-based morphometry and graph theoretical analysis of diffusion MRI and tractography data), which may also hold additional promise for improving prediction of depression symptom severity after childhood TBI (Watson et al., 2019). Functional MRI studies are also required to better understand the mechanisms underlying the prospective relationships identified in this study. For instance, functional MRI studies may help to identify potential aberrancies in inter- and intra-network connectivity that may underlie executive dysfunction and post-injury depression symptoms in the cTBI population (Rausa et al., 2020). Finally, the inclusion of an orthopaedic-injury control group in future research would also help to clarify whether elevated depressive symptoms and executive dysfunction in our TBI sample is unique to individuals with a history of neurological trauma (i.e., a brain-injury–specific effect) or whether these long-term outcomes are explained at least in part by pre-existing risk factors (e.g., premorbid psychiatric problems) that are shared by children who sustain orthopaedic and neurological trauma.

## 5 Conclusions

In addressing a dearth of longitudinal prospective research regarding the link between cTBI and elevated risk of post-injury depressive symptoms, this study identifies neurostructural and neurocognitive risk factors that might help to explain this association in a relatively large sample of children with TBI. As expected, the TBI group showed significantly higher depressive symptoms than TD controls, as well as altered brain morphometry in neural networks implicated in EF. Interestingly, the TBI group also displayed significantly lower EF, which fully mediated the prospective association between altered CEN morphometry and elevated post-injury depressive symptoms in the TBI group. The findings suggest that therapeutic interventions targeting everyday executive function skills, especially emotional regulation and cognitive flexibility, may help in mitigating depressive symptom risk in children with TBI.

## 6 Institutional review board statement

All subjects gave their informed consent for inclusion before they participated in the study. The study was conducted in accordance with the Declaration of Helsinki, and the protocol was approved by the Ethics Committee of The Royal Children’s Hospital (Project No. HREC 64840).

## 7 Informed consent statement

Informed consent was obtained from all subjects involved in the study.

## 8 Data availability statement

The data presented in this study are available on request from the corresponding author. The data are not publicly available due to local ethics requirements.

## Funding

This research received funding from the Victorian Neurotrauma Initiative (VNI), Grant Number COE931.

## CRediT authorship contribution statement

**Nicholas P. Ryan:** Conceptualization, Data curation, Investigation, Project administration, Supervision, Writing – original draft, Writing – review and editing. **Dawn Koester:** Conceptualization, Project administration, Writing – original draft, Writing – review & editing. **Louise Crossley:** Data curation, Investigation, Methodology, Project administration, Resources, Writing – review & editing. **Edith Botchway:** Conceptualization, Project administration, Resources, Writing – review & editing. **Stephen Hearps:** Formal analysis, Investigation, Resources, Writing – review & editing. **Cathy Catroppa:** Conceptualization, Funding acquisition, Investigation, Methodology, Supervision, Writing – original draft, Writing – review & editing. **Vicki Anderson:** Conceptualization, Funding acquisition, Investigation, Methodology, Supervision, Writing – review & editing.

## Declaration of competing interest

The authors declare that they have no known competing financial interests or personal relationships that could have appeared to influence the work reported in this paper.

## Data Availability

Data will be made available on request.
