# Supplementary material for: Delineating the impact of childhood traumatic brain injury (TBI) on long-term depressive symptom severity: Does sub-acute brain morphometry prospectively predict 2-year outcome?
Source: Neuroimage Clin. 2024 Jan 9;41:103565. doi: 10.1016/j.nicl.2024.103565 (PMC10831307; doi:10.1016/j.nicl.2024.103565)
Supplement: Supplementary data 1 [file mmc1.docx]

**Table S1**. Evaluating indirect effects of default mode network (DMN) and mentalizing network (MN) morphometry on depression symptom severity via executive function.

|  | | Indirect effects* | | | | |
| --- | --- | --- | --- | --- | --- | --- |
|  | | Effect | | | Lower CI | Upper CI |
| **CBCL Withdrawn-Depressed** | |  | | |  |  |
| *Model 1: DMN* | |  | | |  |  |
| EF Composite | | -0.013 | | | -0.035 | 0.003 |
| *Model 2: DMN* | |  | | |  |  |
| BRIEF BRI | | -0.004 | | | -0.016 | 0.006 |
| *Model 3: MN* | |  | | |  |  |
| EF composite | | -0.012 | | | -0.029 | 0.001 |
| *Model 4: MN* | |  | | |  |  |
| BRIEF BRI | | -0.004 | | | -0.014 | 0.004 |
| **CBCL Internalising Total** | |  | | |  |  |
| *Model 1: DMN* | |  | | |  |  |
| EF Composite | | -0.046 | | | -0.122 | 0.011 |
| *Model 2: DMN* | |  | | |  |  |
| BRIEF BRI | | -0.024 | | | -0.089 | 0.036 |
| *Model 3: MN* | |  | | |  |  |
| EF composite | | -0.044 | | | -0.112 | 0.001 |
| *Model 4: MN* | |  | | |  |  |
| BRIEF BRI | | -0.023 | | | -0.082 | 0.026 |
| *Note: A 95% confidence interval (CI) including zero denotes a non-significant indirect effect. |  | |  |  | | |
