# Supplementary material for: Delineating the impact of childhood traumatic brain injury (TBI) on long-term depressive symptom severity: Does sub-acute brain morphometry prospectively predict 2-year outcome?
Source: Neuroimage Clin. 2024 Jan 9;41:103565. doi: 10.1016/j.nicl.2024.103565 (PMC10831307; doi:10.1016/j.nicl.2024.103565)
Supplement: Supplementary data 2 [file mmc2.docx]

**Table S2.** Sensitivity analysis: indirect effects of CEN morphometry on depression symptom severity, after exclusion of severe TBI cases.

|  | | Indirect effects | | | | |
| --- | --- | --- | --- | --- | --- | --- |
|  | | Effect | | | Lower CI | Upper CI |
| **CBCL Withdrawn-Depressed** | |  | | |  |  |
| Model 1: CEN | |  | | |  |  |
| EF Composite* | | -0.017 | | | -0.041 | -0.001 |
| Model 2: CEN | |  | | |  |  |
| BRIEF BRI* | | -0.009 | | | -0.018 | -0.002 |
| **CBCL Internalising Total** | |  | | |  |  |
| Model 1: CEN | |  | | |  |  |
| EF composite* | | -0.068 | | | -0.158 | -0.007 |
| Model 2: CEN | |  | | |  |  |
| BRIEF BRI* | | -0.053 | | | -0.100 | -0.011 |
| *Significant indirect effect |  | |  |  | | |
